# A meta-analysis to examine whether nitrification inhibitors work through selectively inhibiting ammonia-oxidizing bacteria

**DOI:** 10.3389/fmicb.2022.962146

**Published:** 2022-07-19

**Authors:** Jilin Lei, Qianyi Fan, Jingyao Yu, Yan Ma, Junhui Yin, Rui Liu

**Affiliations:** College of Resources and Environmental Sciences, China Agricultural University, Beijing, China

**Keywords:** nitrous oxide, DMPP, ammoxidation, edaphic conditions, microorganism

## Abstract

Nitrification inhibitor (NI) is often claimed to be efficient in mitigating nitrogen (N) losses from agricultural production systems by slowing down nitrification. Increasing evidence suggests that ammonia-oxidizing archaea (AOA) and ammonia-oxidizing bacteria (AOB) have the genetic potential to produce nitrous oxide (N_2_O) and perform the first step of nitrification, but their contribution to N_2_O and nitrification remains unclear. Furthermore, both AOA and AOB are probably targets for NIs, but a quantitative synthesis is lacking to identify the “indicator microbe” as the best predictor of NI efficiency under different environmental conditions. In this present study, a meta-analysis to assess the response characteristics of AOB and AOA to NI application was conducted and the relationship between NI efficiency and the AOA and AOB *amo*A genes response under different conditions was evaluated. The dataset consisted of 48 papers (214 observations). This study showed that NIs on average reduced 58.1% of N_2_O emissions and increased 71.4% of soil $NH{}_{4}^{+}$ concentrations, respectively. When 3, 4-dimethylpyrazole phosphate (DMPP) was applied with both organic and inorganic fertilizers in alkaline medium soils, it had higher efficacy of decreasing N_2_O emissions than in acidic soils. The abundance of AOB *amo*A genes was dramatically reduced by about 50% with NI application in most soil types. Decrease in N_2_O emissions with NI addition was significantly correlated with AOB changes (*R*^2^ = 0.135, *n* = 110, *P* < 0.01) rather than changes in AOA, and there was an obvious correlation between the changes in $NH{}_{4}^{+}$ concentration and AOB *amo*A gene abundance after NI application (*R*^2^ = 0.037, *n* = 136, *P* = 0.014). The results indicated the principal role of AOB in nitrification, furthermore, AOB would be the best predictor of NI efficiency.

## Introduction

Nitrification is a crucial process in the nitrogen (N) cycle, involving the oxidization of ammonium ($NH{}_{4}^{+}$) to nitrate ($NO{}_{3}^{-}$) through nitrite ($NO{}_{2}^{-}$). The process supplies significant amounts of N to be taken up by growing crops. However, unabsorbed N is lost to the atmosphere or the soil below the root zone. These unwanted losses of N have significant implications for the environment, for example $NO{}_{3}^{-}$ leaching and greenhouse gas emissions (GHG), particularly nitrous oxide (N_2_O). N_2_O is a potent GHG which greatly contributes to global climate change, it has a 265-fold higher global warming potential than CO_2_ (IPCC, 2014) and it is involved in the destruction of the protective ozone layer (Ravishankara et al., 2009), which has become one of society’s most important challenges (Desloover et al., 2012).

The application of nitrification inhibitor (NI) is a promising technology to reduce N losses in different kinds of soil systems. In agriculture, several chemical compounds were designed to delay the steps of microbial oxidation of $NH{}_{4}^{+}$ to $NO{}_{3}^{-}$ in the soil to decrease N_2_O emissions, such as 3, 4-dimethylpyrazole phosphate (DMPP), dicyandiamide (DCD), and 2-chloro-6-(trichloromethyl) pyridine (nitrapyrin). Of these, DMPP is the most efficient commercial compound, which is applied as dihydrogen phosphate salt to reduce its loss through evaporation. NIs target the first step, i.e., the enzyme ammonia monooxygenase (AMO) in the case of DMPP (and other N-containing inhibitors) presumably through reversible complexation of the enzyme’s Cu center (McCarty, 1999; Beeckman et al., 2018). Ammonia-oxidizing archaea (AOA) and bacteria (AOB) both perform the first step of nitrification and are probably targets for NIs. The impact of NIs in delaying nitrification and reducing N_2_O emissions has been widely reported (Huang et al., 2014; Cai and Akiyama, 2017; Xu et al., 2019). However, the effectiveness of NIs varies greatly within different soils (Shi et al., 2017; Zhu et al., 2019), fertilizers (Pereira et al., 2010), and moisture content (Chen et al., 2010; Hu et al., 2015a; Fan et al., 2019). Soil temperature is another key factor controlling NI efficiency, which can subside after 1 week at 35°C (Barth et al., 2008; Chen et al., 2015). Furthermore, many studies focused on the impact and contribution of soil microorganisms on N_2_O emissions (Chen et al., 2019; Lazcano et al., 2021; Yang et al., 2021; You et al., 2022). However, there is still a lack of direct evidence on whether soil microbial community, especially AOA and AOB, affects NI efficacy (Chen et al., 2010; Guardia et al., 2018; Lam et al., 2018).

Within the major N-cycling microbes, AOA and AOB are important functional strains, and both carry the *amo*A gene which encodes AMO (Xia et al., 2018). Due to its strong conserved nature, the *amo*A gene is often used as a biomarker for exploring ammonia-oxidizing microorganisms (Schleper and Nicol, 2010). This has certain advantages in analyzing the genetic diversity of ammonia-oxidizing microorganisms. The differences in cellular biochemistry and physiology between AOA and AOB lead to their different ecological niches in different agroecosystems in terms of sensitivity to soil pH, soil texture, N forms, moisture, temperature and other conditions (Morimoto et al., 2011; Prosser and Nicol, 2012; Hu et al., 2015b). Hu et al. (2013) showed that the increase of nitrification activity in most acidic soils was positively correlated with the increase of AOA quantity, but not with AOB. In general, AOB dominates nitrification in neutral and alkaline soils, while AOA is more suitable to the acidic environment (Lu et al., 2012; Li et al., 2018). Increasing the $NH{}_{4}^{+}$ concentration will enhance the nitrification activity of AOB (Okano et al., 2014), while AOA prefers an environment with a lower $NH{}_{4}^{+}$ concentration. For example, a low pH environment is favorable for the formation of $NH{}_{4}^{+}$ and changes the utilization of $NH{}_{4}^{+}$ by AOB (Ying et al., 2017). Therefore, different edaphic and environmental conditions would influence AOA and AOB nitrification activity, and in turn affect the response of AOA and AOB to NI application.

To date, most studies on the inhibitory effect of NIs on AOA and AOB have focused either on the change of the *amo*A gene population (Prosser and Nicol, 2008; Kleineidam et al., 2011) or on the change of the AOA and AOB community (Zhang et al., 2012a; Liu et al., 2015). There is very little research available with respect to the “indicator microbe,” AOA or AOB, as the best predictor of NI efficiency under different environmental conditions. In acidic soils, AOA played a dominant role in nitrification and N_2_O production (Liu et al., 2016; Gu et al., 2019; Zhou et al., 2020), but NIs especially DMPP showed a lower inhibitory effect in acidic soils. Furthermore, many studies showed that NIs effectively decreased the AOB population, but not AOA (Gong et al., 2013; Liu et al., 2015, 2017; Dong et al., 2018; Yin et al., 2021). Hence, it was hypothesized that AOB are more sensitive to NIs than AOA and NIs would work through selectively inhibiting AOB.

Using a meta-analytical approach, results of 48 individual studies were combined to estimate the variations of NI efficiency in different edaphic and experimental conditions. Moreover, the general trends in the response of AOA and AOB abundance to NI addition were explored. Lastly, the efficiency of NIs was investigated by looking at the relationship among AOA or AOB *amo*A gene abundance and N_2_O emissions. This approach will help to identify the “indicator microbe,” AOA or AOB, as the best predictor of NI efficiency under different environmental conditions.

## Materials and methods

### Data compilation

**FIGURE 1:**
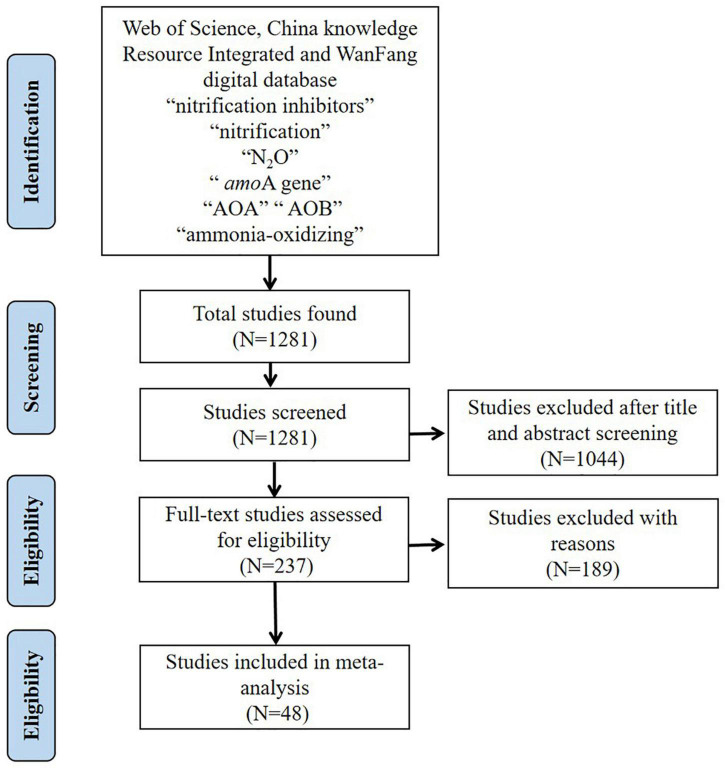
Selection of studies for inclusion in the meta-analysis.

The databases used for the data collection included Web of Science, WanFang digital database and China knowledge Resource Integrated to search for relevant studies published between 2010 and 2021. The key search terms were: nitrification inhibitors, nitrification, N_2_O, *amo*A gene, AOA, AOB, ammonia-oxidizing. The number of studies selected at various stages is shown in the flow diagram in Figure 1. After screening the literature, the database consisted of 214 selected pairwise comparisons reported in 48 studies (Supplementary Table 1), which met predetermined quality criteria (studies with replication, with detailed information, and performed under greenhouse, field and controlled laboratory conditions) (Abalos et al., 2022). All the studies included pairwise comparisons in which treated soil (with NI addition) was compared to an untreated control (without NI addition). Furthermore, the collated observations which were screened should measure the abundance of the *amo*A functional gene for AOB and AOA and the studied ecosystem type belonged to pastoral or agricultural environments.

In these present analyses, to take full advantage of published results, multiple experimental treatments from the same study were included (e.g., treatments that varied by N fertilizer type). However, only one measurement from each experimental replicate was included to maximize independence among measurements (Carey et al., 2016). For instance, the highest $NH{}_{4}^{+}$ concentration was selected from the studies where $NH{}_{4}^{+}$ concentration was measured multiple times from the same treatment.

The mitigation of cumulative N_2_O emissions and the changes in $NH{}_{4}^{+}$ concentrations were considered as the evaluation variables of the NI inhibitory effect. The change of *amo*A gene abundance was reflected as the influence of NIs on microorganisms. Of all the 214 observations in the present study, observations 155, 174, 166, 147 concerned N_2_O yield, $NH{}_{4}^{+}$ concentration, AOB *amo*A and AOA *amo*A gene abundance, respectively. Data on soil physical and chemical properties and experimental conditions were also collected from the original literature to analyze their influence on NI efficacy. Soil pH, soil organic matter (SOM), soil texture, soil moisture content (water filled pore space, WFPS and water holding capacity, WHC), soil temperature (TEMP), N fertilizer type, N application rate (NR), and NIs type were chosen to assess how edaphic conditions and management measures influenced NI efficacy. The following were the categorical variables classified into different groups:

– Soil pH: (1) soil pH ≤ 6, (2) 6 < soil pH < 8, (3) soil pH ≥ 8
– SOM (g kg^–1^): (1) SOM ≤ 20, (2) 20 < SOM ≤ 40, (3) SOM > 40
– Soil texture: (1) coarse (sand, loamy sand, sandy loam, loam, silt loam, and silt), (2) medium (sandy clay loam, clay loam, and silty clay loam), (3) fine (sandy clay, silty clay, and clay)
– WFPS and WHC: (1) WFPS and WHC ≤ 40%, (2) 40% < WFPS and WHC ≤ 60%, (3) 60% < WFPS and WHC ≤ 80%, (4) WFPS and WHC > 80%
– TEMP (°C): (1) TEMP ≤ 20, (2) 20 < TEMP ≤ 25, (3) TEMP > 25
– N fertilizer type: (1) $NH{}_{4}^{+}$ based fertilizer (including ammonium chloride (NH_4_Cl), ammonium nitrate (NH_4_NO_3_) and ammonium sulfate [(NH_4_)_2_SO_4_)], (2) organic fertilizer (including livestock manure and urine), (3) urea, (4) both (combination of organic and inorganic fertilizer)
– NR (kg N ha^–1^): (1) NR ≤ 100, (2) 100 < NR ≤ 150, (3) NR > 150
– NIs type: (1) DMPP, (2) DCD, (3) nitrapyrin and others

### Data analysis

The natural logarithmic response ratio (ln*RR*) as an effect size for each observation was calculated as Equation (1) (Luo et al., 2006):


(1) $$\ln RR=ln\frac{Xt}{Xc}=\ln Xt-\ln Xc$$

where *Xt* is the average value of index *X* from NIs treatments and *Xc* is the average from the control treatments.

The results were expressed by using the conversion equation according to Equation (2) as percentage change:


(2) $$\%\text{change}=\left(e^{\ln RR}-1\right)\times 100$$

A positive percentage change indicated increases in N_2_O yield, $NH{}_{4}^{+}$ concentration, and *amo*A gene abundance after NI addition, while a negative percentage change indicated decreases in these variables (Sha et al., 2020). Replication-based weighting was used to avoid the effect of extreme weightings, using the following Equation (3) (Groenigen et al., 2011):


(3) $$\text{W}=\frac{n_{t}\times n_{c}}{n_{t}+n_{c}},\text{V}=\frac{1}{W}=\frac{n_{t}+n_{c}}{n_{t}\times n_{c}}$$

where *n*_*t*_ and *n*_*c*_ were the number of replications in the treatment group and control group, respectively.

The mean effect size of environmental and management variables on NI efficacy was calculated by a random-effect model and 95% of confidence intervals (CIs) were produced by a bootstrapping procedure with 4,999 iterations (Sha et al., 2020). In the present meta-analysis, Metawin 2.1 software (Rosenberg et al., 2000) was applied to perform all the calculations. If the 95% CIs did not overlap zero, the effects of NIs on the evaluation variables were considered significant. When the 95% CIs of each categorical group did not overlap, there were significantly different from each other. For each categorical variable, total heterogeneity (Q_t_) was segmented into within-group (Q_*w*_) and between-group (Q_b_). Q_b_/Q_t_ describes the proportion of total variation explained by each modifier. The *P*-value is the probability value for randomization tests (999 permutations) with sample size as the weighting function, calculated only for the Q_b_ values. A particular categorical variable was considered to have a significant impact on the response ratio when Q_b_ was significant (*P* < 0.05) and was larger than the critical value (Carey et al., 2016). The heterogeneity in different categorical groups for each explanatory variable was also reported in Tables 1, 2. Of all observations (from the 48 studies) included in this meta-analysis, 113 and 110 measured the effect of NIs on N_2_O emissions, and AOA and AOB *amo*A gene abundance simultaneously. Of those, 133 and 136 measured NI effects on $NH{}_{4}^{+}$ concentration changes in addition to AOA and AOB *amo*A gene abundances, respectively. Based on these observations, a regression analysis was conducted in Origin 9.0 to explore the relationship between the effects of NIs on $NH{}_{4}^{+}$ concentration, N_2_O emission and *amo*A gene abundance.

**TABLE 1:** Between-group heterogeneity (Q_b_) illustrating the effects of NIs additions on N_2_O emission and $NH{}_{4}^{+}$ concentration across categorical modifiers.

Explanatory variables	N_2_O		$NH{}_{4}^{+}$	
	Q_b_	Q_b_/Q_t_	Q_b_	Q_b_/Q_t_
Soil pH	16.06**	0.09	3.11	0.02
Soil texture	24.82**	0.16	0.64	0.006
Soil organic matter	3.45	0.03	15.02**	0.11
Moisture	2.60	0.02	9.15*	0.06
Temperature	3.81	0.02	18.99**	0.10
N application rate	3.47	0.02	13.30**	0.08
Fertilizer type	9.71*	0.06	7.24	0.04
NIs type	2.63	0.02	6.28*	0.04

Q_b_/Q_t_ describes the proportion of total variation explained by each modifier. The P-value is the probability value for randomization tests (999 permutations) with sample size as the weighting function, calculated only for the Q_b_ values; *P < 0.05; **P < 0.01.

**TABLE 2:** Between-group heterogeneity (Q_b_) illustrating the effects of NIs additions on ammonia oxidizer across categorical modifiers.

Explanatory variables	AOB		AOA	
	Q_b_	Q_b_/Q_t_	Q_b_	Q_b_/Q_t_
Soil Ph	3.53	0.03	0.16	0.002
Soil texture	1.37	0.01	5.99*	0.10
Soil organic matter	2.04	0.02	2.63	0.06
Moisture	3.31	0.02	1.39	0.02
Temperature	3.06	0.02	0.50	0.007
N application rate	0.89	0.006	3.00	0.04
Fertilizer type	43.76**	0.22	1.86	0.02
NIs type	0.80	0.01	0.17	0.002

Q_b_/Q_t_ describes the proportion of total variation explained by each modifier. The P-value is the probability value for randomization tests (999 permutations) with sample size as the weighting function, calculated only for the Q_b_ values; *P < 0.05; **P < 0.01.

## Results

### Inhibitory effect of nitrification inhibitors on nitrous oxide emissions

**FIGURE 2:**
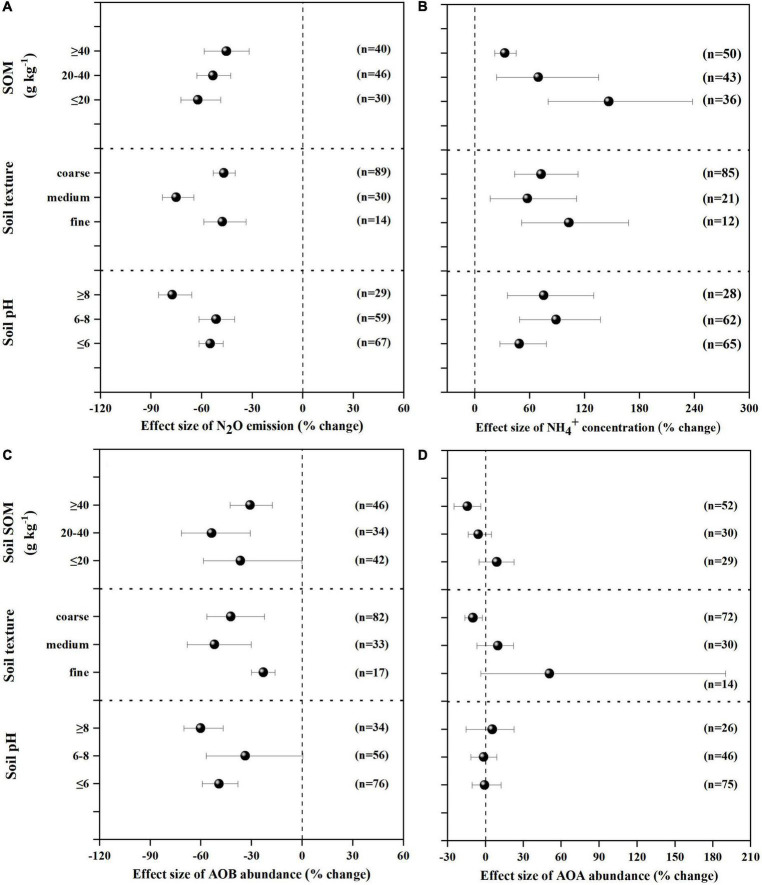
Mean response ratios (% change) and bootstrapped 95% Confidence Intervals (CI) for the effects of soil properties on the N_2_O emissions **(A)**, $NH{}_{4}^{+}$ concentration **(B)**, AOB gene abundance **(C)** and AOA gene abundance **(D)** after NIs application. Values in parentheses represent the number of observations.

**FIGURE 3:**
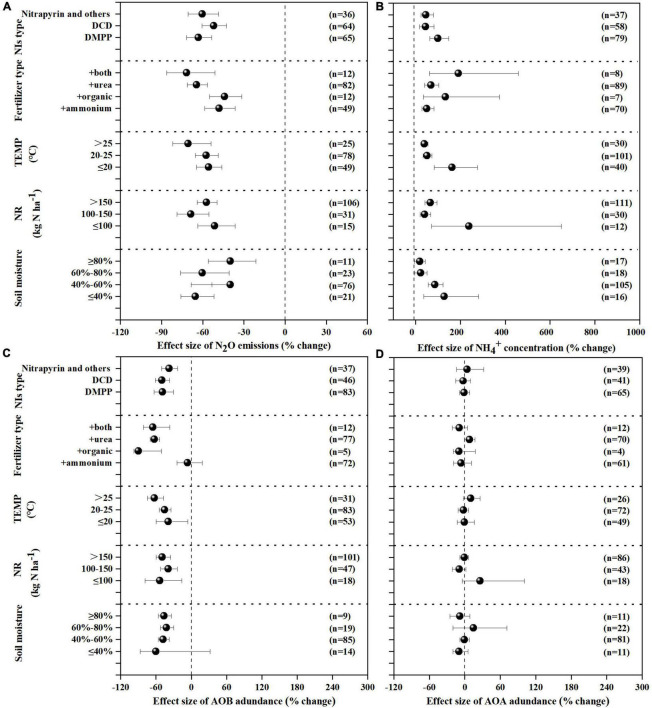
Mean response ratios (% change) and bootstrapped 95% Confidence Intervals (CI) for the effects of experiment conditions on the response of N_2_O **(A)**, $NH{}_{4}^{+}$ **(B)**, AOB **(C)**, AOA **(D)** after NIs treatment. Values in parentheses represent the number of observations.

NIs effectively decreased N_2_O emissions across all experimental and edaphic conditions. For the efficacy of NIs, soil pH, soil texture and fertilizer type were the best explanatory variables (Table 1 and Figures 2A, 3A). N_2_O emissions were reduced by 54.9, 51.4, 77.4% by NIs in acidic, neutral and alkaline soils, respectively (Figure 2A), indicating that NIs performed better in alkaline soils than in neutral and acidic soils. The efficacy of NIs on reducing N_2_O emissions reached 75.2% in medium soil (Figure 2A, 95% CIs ranged 8.0–10.62%) while it only reached 46.9 and 47.7% in coarse and fine soils, respectively. The combined application of NIs with both organic and $NH{}_{4}^{+}$ fertilizer or urea (at a relatively high N rate above 100 kg N ha^–1^) performed better (71.7%) than the combined application of NIs with organic or inorganic fertilizer alone (43.8 and 52.2%) (Figure 3A). Of all observations, DMPP was the best NI to mitigate N_2_O emissions (63.3%).

### Effect of nitrification inhibitors on $NH{}_{4}^{+}$ concentration

$NH{}_{4}^{+}$ concentration was increased by 71.4% on average with NI application across all experimental and edaphic conditions. NIs had a stronger ability to restrain the oxidization of $NH{}_{4}^{+}$ in soil with low SOM (below 20 g kg^–1^) when the soil WHC/WFPS was lower than 40% (Figures 2B, 3B). The effect of NIs in slowing nitrification was better when the temperature was lower than 20°C (Figure 3B and Table 1, *P* < 0.01). Different NIs showed different efficacies in inhibiting nitrification, and DMPP was the most effective inhibitor compared with others (98.9% average change, 95% CIs range 36.5–48.8%, Figure 3B and Table 1, *P* < 0.05). The greater soil $NH{}_{4}^{+}$ retention by DMPP was associated with a lower N application rate (below 100 kg N ha^–1^) (Figure 3B and Table 1, *P* < 0.05). In addition, $NH{}_{4}^{+}$ concentration in alkaline and neutral soils was more responsive to NI addition than in acid soils.

### Effect of nitrification inhibitors on ammonia-oxidizing bacteria and archaea

AOB *amo*A gene abundance negatively responded to NI addition (Figures 2C, 3C). The response ratio was always lower than or equal to zero, the magnitude significantly depended on fertilizer type (*P* < 0.01; Table 2). The efficacy of NIs on reducing AOB *amo*A gene abundance reached up to 90.08% when NIs were applied with organic fertilizer, which was higher than in NI application combined with inorganic fertilizer alone and with both organic and inorganic fertilizers (Figure 3C). However, no significant differences were observed in the response of AOB gene abundance to NIs across most of the categorical variables (*P* > 0.05; Table 2), including soil pH, NI type, SOM, moisture, TEMP, and NR.

The response ratio of AOA *amo*A gene abundance was always slightly lower than or equal to zero. It was observed that only soil texture had significant impact on the responses of AOA to NIs (*P* < 0.05; Table 2). Under certain experimental and edaphic conditions, NIs increased AOA *amo*A gene abundances (Figures 2D, 3D). Notably, AOA *amo*A gene abundance positively responded to NIs in medium and fine soils (*P* < 0.05; Table 2). Furthermore, when soil moisture was between 60 and 80% WHC/WFPS or NR was below 100 kg N ha^–1^, NIs could increase AOA *amo*A gene abundance (Figure 3D).

### Relationship between nitrous oxide emissions, the efficiency of nitrification inhibitors and amoA gene response

**FIGURE 4:**
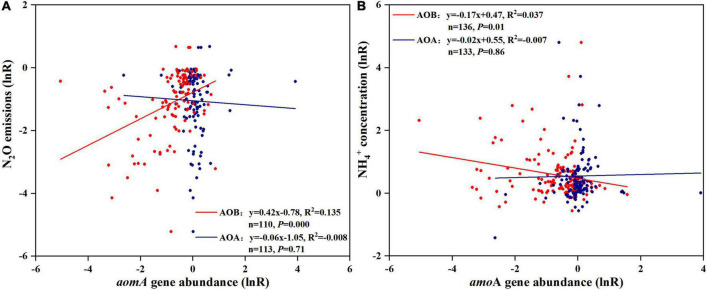
Relationship between effect size (lnR) of N_2_O emissions **(A)** or $NH{}_{4}^{+}$ concentration **(B)** and effect size (lnR) of AOA and AOB *amo*A gene abundance. Line is the best-fit regression, where AOB-NIs effectiveness is the red line and AOA-NIs effectiveness is the blue line. Each symbol represents one observation; red point, AOB, blue point, AOA.

The response ratio of AOB was significantly and positively correlated with N_2_O emissions (N_2_O emission[lnR] = 0.42 × AOB[lnR]−0.78, *R*^2^ = 0.14, *P* < 0.01; Figure 4A). In contrast, there was no significant correlation observed between the response ratio of AOA and N_2_O emissions (N_2_O emission[lnR] = −0.06 × AOA[lnR]−1.05, *R*^2^ < 0.00, *P* = 0.71; Figure 4A). There was an obvious correlation between the changes in $NH{}_{4}^{+}$ concentration and AOB *amo*A gene abundance after NI application ($NH{}_{4}^{+}$ concentration[lnR] = −0.17 × AOB[lnR] + 0.47, *R*^2^ = 0.04, *P* = 0.014; Figure 4B).

## Discussion

### Effect of edaphic and experimental conditions on nitrification inhibitor efficacy

Soil pH was an important explanatory variable for NI efficacy in reducing N_2_O emissions (Cui et al., 2021). The results from the current meta-analysis showed that NIs had different effects on decreasing N_2_O emissions under different soil pH (Figure 2A and Table 1, *P* < 0.01), and NIs efficacy had a positive response to soil pH. Firstly, it may be attributed to NIs being retained for longer in alkaline soils (pH ≥ 8). Soil pH has been considered as one of the most important factors controlling NI efficacy, because pH has potential to impact the degradation rate of the NIs in soils. Cui et al. (2021) showed that DMPP performed better in alkaline soil compared to acid soil conditions, which may be caused by the shorter half-life time of DMPP in acidic soil compared to alkaline soil. DMPP undergoes degradation in soil through chemical reaction steps, potentially involving reactive oxygen species (ROS) generated through abiotic and/or biotic processes (Sidhu et al., 2021), which would possibly be affected by pH. Secondly, soil pH played a vital role in controlling N_2_O emissions from soils (Morkved et al., 2007). Wang Y. et al. (2018) demonstrated that soil pH was negatively correlated with N_2_O emission, indicating less N_2_O emission from alkaline soils. In the current research, the inhibition efficacy of NIs on reducing N_2_O emissions increased with soil pH, indicating that NIs were more effective in alkaline soils. It may be also attributed to the less N_2_O emissions from alkaline soils.

SOM and soil texture were also considered as main factors affecting NI performance (Jarvis et al., 2007). Previous studies have reported a negative correlation between NI efficacy and SOM and clay content (Barth et al., 2008; Zhu et al., 2019). High SOM and clay content could easily adsorb NIs, which would influence their availability and effectiveness (Zhang et al., 2020; Cui et al., 2021). Furthermore, SOM could be used by soil microorganisms as energy, carbon (C), and N source, which improve microbial bioactivity, leading to accelerated biodegradation of NIs (Fisk et al., 2015). Clay had a protective effect on nitrifying oxidizers (Neufeld and Knowles, 1999). Higher clay content might make microorganisms less susceptible to being affected by inhibitors, thus weakening NIs inhibitory effects. Therefore, the current study found that NIs delayed ammonia oxidation and inhibited N_2_O emissions more efficiently in medium soils with lower SOM.

Soil temperature had a significant effect on NIs inhibition on nitrification (*P* < 0.01). Temperature influenced the rate of nitrification, which might affect the inhibitory effect of NIs on nitrification and $NH{}_{4}^{+}$ retention (Mathieu et al., 2006). Irigoyen et al. (2003) reported that the nitrification rate accelerated at 20°C, but slowed down when the temperature reached up to 30°C. A lower temperature (≤ 20°C) was favorable for improving the efficacy of NIs on delaying the nitrification rate, which may also be attributed to the rapid decomposition of NIs by microorganisms in high temperature (Irigoyen et al., 2003; Wang X. et al., 2018). Yu et al. (2015) found that the increased $NH{}_{4}^{+}$ concentration by DMPP at 15°C was 56 times higher than that at 25°C due to better persistence of the molecule of DMPP at 15°C. Hauser and Haselwandter (1990) also demonstrated that the degradation rate of DCD reached its highest between 25°C and 33°C. The above studies were consistent with the results in this study, in which the addition of NIs increased $NH{}_{4}^{+}$ concentration in temperatures below 20°C.

Kirschke et al. (2019) found that the effect of NIs on nitrification was negatively correlated with soil moisture, which was consistent with this study. The probable reason was that higher water content may increase the distance between NI and $NH{}_{4}^{+}$ because of faster diffusion of $NH{}_{4}^{+}$ than that of NIs (Azam et al., 2001). On the other hand, the soil was supposed to be hypoxic at high water content (80% WFPS), inducing denitrification occurrence and dominance (Menéndez et al., 2008). Nitrification dominated at 40% WFPS, which was conducive to the effect of NIs on $NH{}_{4}^{+}$ retention (Menéndez et al., 2012). This would also explain the negative correlation between the effect of NIs on $NH{}_{4}^{+}$ retention and soil moisture in the current study.

The combined application of NIs with the appropriate N fertilizers could improve their efficacy (Vinzent et al., 2018). The present results showed that the combined application of NIs with organic fertilizer could enhance NIs inhibitory effect on N_2_O emissions. On the one hand, the application of organic fertilizer significantly improved soil pH, which could prolong the retention time of NIs and thus improve the efficacy of NIs in inhibiting N_2_O emissions (Zhang et al., 2012b). On the other hand, as observed in the current study, the efficacy of NIs in reducing AOB *amo*A gene abundance was highest when NIs were applied with organic fertilizer, thus N_2_O emission mitigation by NIs reaching its maximum. NR significantly influenced the effect size of NIs on $NH{}_{4}^{+}$ retention. Better NI efficacy in increasing $NH{}_{4}^{+}$ concentration could be observed at a lower N application rate (≤100 kg N ha^–1^). This is in accordance with previous findings by Rowlings et al. (2016), which revealed that N application which was less than the conventional rate could increase DMPP performance. Inappropriate N application rates may result in a large N surplus, providing adequate substrate of $NH{}_{4}^{+}$ for ammonia volatilization and thus reducing the efficacy of the NI in increasing $NH{}_{4}^{+}$ concentration (Nauer et al., 2018).

### Response of ammonia oxidizers to nitrification inhibitors

AOB *amo*A gene abundance negatively responded to NI addition under different edaphic and experimental conditions. However, in contrast to AOB, AOA *amo*A gene abundance responded positively to NI addition in medium and fine soils. Fan et al. (2019) found an increase in AOA abundance after DMPP application in the tested soils, which was consistent with our results. Our results were also in good agreement with the study by Hink et al. (2018) and Fan et al. (2022), which reported that AOA growth were accelerated while AOB were inhibited with NIs. The growth of AOA might be promoted by organic compounds (Tourna et al., 2011; Ai et al., 2013), and it is possibly because that NIs such as acetylene and DMPP could be available C substrates for AOA (Florio et al., 2014; Hink et al., 2017). Compared to coarse soils, fine and medium soils showed a generally higher accumulation potential of SOM which provided sufficient C and N substrates for AOA proliferation (Kögel-Knabner et al., 2008; Dieckow et al., 2009). In line with results of this study, Shen et al. (2013) illustrated that most of the NIs appeared to have no effect on AOA in agricultural soils. Shi et al. (2016) also discovered that DMPP could strongly influence the metabolic activity of AOB by using DNA-stable isotope probing (SIP) but did not influence AOA. The potential physiological or metabolic differences between AOA and AOB (Prosser and Nicol, 2012) may explain the different responses of AOB and AOA to NIs. Furthermore, the most commonly used inhibitors suppressed microbial activity by chelating Cu active sites in AMO, and the periplasmic *Amo*B, a subunit of ammonia monooxygenase, presumably contains a copper-catalyzed active site (Monaghan et al., 2013; Beeckman et al., 2018). Lawton et al. (2014) found that archaea *Amo*B is a non-active enzyme and NIs tend to chelate on the active site of AOB to inhibit its activity, which indicated that the structural difference of the *Amo*B subunit and the ecophysiological differences also possibly lead to the variation in sensitivity among AOA and AOB to NIs (Tolar et al., 2017).

Fertilizer forms significantly affected the response of AOB to NIs, rather than the response of AOA (Tao et al., 2017). The results from the current study demonstrated that NIs showed the best performance in slowing down AOB growth in the case of organic fertilizer application, however, there was no difference observed on AOA abundance with NI application under different fertilizer forms. Wang et al. (2014) found an obvious stimulating effect of manure fertilization on the efficacy of NIs in reducing the population of AOB rather than AOA in a paddy soil, which was confirmed by the results of the current analysis. The application of organic fertilizer would provide an ideal alkaline environment for NIs to reduce AOB *amo*A gene abundance, which may be attributed to better activity and sensitivity of AOB to NI addition under alkaline conditions. But AOA adapted to low pH conditions (i.e., have a pH optima below 7; Hatzenpichler, 2012).

The best-fit regression in this study showed that N_2_O mitigation and $NH{}_{4}^{+}$ concentration increase by NIs was positively correlated with the decrease of AOB-*amo*A gene abundance by NI application but not AOA-*amo*A. This supported the hypothesis that AOB are more sensitive to NIs than AOA and NIs would work through selectively inhibiting AOB. Previous studies illustrated that although the number of AOA far exceeds that of AOB in most terrestrial ecosystems, the N_2_O production capacity of AOB was 10–1,000 times higher than that of AOA (Leininger et al., 2006; Jung et al., 2011; Xia et al., 2011; Gu et al., 2019). The main reason for that was that AOB-related N_2_O was produced *via* nitrifier-denitrification and incomplete NH_2_OH oxidation (Shaw et al., 2006; Wu et al., 2018), while the N_2_O produced by AOA could not be attributed to nitrifier-denitrification, due to a lack of NO reductase (Tourna et al., 2011; Jung et al., 2014; Stieglmeier et al., 2014). Kozlowski et al. (2016) showed direct evidence that N_2_O produced by AOA was attributed to abiotic reactions of released NO under anoxic conditions, in which Nitrososphaera viennensis EN76(T) (a Thaumarchaeon) was used as a test AOA. There was an obvious correlation between $NH{}_{4}^{+}$ and AOB (*P* < 0.05; Figure 4B), indicating the high inhibitory effect of NIs on nitrification through inhibiting AOB, which was consistent with the results reported by Zerulla et al. (2001) and Di and Cameron (2011). The obvious correlation between $NH{}_{4}^{+}$ concentration and AOB also revealed the dominate role of AOB in nitrification. Although the relationship between AOA *amo*A gene abundance and N_2_O emissions, $NH{}_{4}^{+}$ concentration after NI application was found to be insignificant in this study, AOA was also important for nitrification in soils. AOA had been shown to play an integral role in soil nitrification of some unmanaged soils (Huang et al., 2011; Isobe et al., 2015), with the greatest contribution likely occurring in N-limited scenarios. As observed in this study, AOB was more sensitive to NIs than AOA, even in soils where AOA were more abundant.

## Conclusion

Soil pH, soil texture, SOM, soil temperature, and N application rate were identified to be the factors most affecting the efficacy of NIs. There was a significant positive correlation between NIs efficacy on decreasing N_2_O emissions, increasing $NH{}_{4}^{+}$ concentration and AOB *amo*A gene abundance reduction after NIs. Taken together, for both soil and experimental conditions, AOB plays a key role in nitrification and NIs specifically inhibit AOB rather than AOA, which indicates AOB would be the best predictor of NI efficiency. These results would provide a scientific basis for better modeling and N management strategies to reduce N_2_O emissions and improve N use efficiency in agricultural systems.

## Data availability statement

The original contributions presented in this study are included in the article/Supplementary Material, further inquiries can be directed to the corresponding author.

## Author contributions

JL: data curation, writing – original draft preparation and review and editing, conceptualization, and validation. QF: methodology, software, and writing – original draft preparation. JiY: formal analysis, investigation, and resources. YM and JuY: writing – reviewing and editing. RL: supervision, project administration, funding acquisition, conceptualization, and writing – reviewing and editing. All authors contributed to manuscript revision, read, and approved the submitted version.

## Conflict of interest

The authors declare that the research was conducted in the absence of any commercial or financial relationships that could be construed as a potential conflict of interest.

## Publisher’s note

All claims expressed in this article are solely those of the authors and do not necessarily represent those of their affiliated organizations, or those of the publisher, the editors and the reviewers. Any product that may be evaluated in this article, or claim that may be made by its manufacturer, is not guaranteed or endorsed by the publisher.
