# Supplementary material for: A meta-analysis to examine whether nitrification inhibitors work through selectively inhibiting ammonia-oxidizing bacteria
Source: Front Microbiol. 2022 Jul 19;13:962146. doi: 10.3389/fmicb.2022.962146 (PMC9343776; doi:10.3389/fmicb.2022.962146)
Supplement: Supplementary file 2 [file Table_1.docx]

Barrena, I., Menendez, S., Correa-Galeote, D., Vega-Mas, I., Bedmar, E.J., Gonzalez-Murua, C., et al. (2017). Soil water content modulates the effect of the nitrification inhibitor 3,4-dimethylpyrazole phosphate (DMPP) on nitrifying and denitrifying bacteria. Geoderma. 303, 1-8. doi: 10.1016/j.geoderma.2017.04.022

Bai, X., Xia, Z., Guo, Y., Zhang, Y., Xu, H., Wang, Z. (2012). Effects of nitrification inhibitors on N2O emission from different upland agricultural soils. Chinese Journal of Ecology. 31(9), 2319-2329. doi: 10.13292/j.1000-4890.2012.0334

Cassman, N.A., Soares, J.R., Pijl, A., Lourenço, K.S., van Veen, J.A., Cantarella, H., et al. (2019). Nitrification inhibitors effectively target N_2_O‐producing Nitrosospira spp. in tropical soil. Environmental Microbiology. 21, 1241-1254. doi: 10.1111/1462-2920

Chen, H., Yin, C., Fan, X., Ye, M., Peng, H., Li, T., et al. (2019). Reduction of N_2_O emission by biochar and/or 3,4-dimethylpyrazole phosphate (DMPP) is closely linked to soil ammonia oxidizing bacteria and *nos*ZI-N_2_O reducer populations. Science of the Total Environment. 694, 133658. doi: 10.1016/j.scitotenv.2019.133658

Cui, P., Fan, F., Yin, C., Li, Z., Song, A., Wan, Y., et al. (2013). Urea- and nitrapyrin-affected N_2_O emission is coupled mainly with ammonia oxidizing bacteria growth in microcosms of three typical Chinese arable soils. Soil Biology and Biochemistry. 66, 214-221. doi: 10.1016/j.soilbio.2013.08.001

Duan, P., Zhang, Q., Zhang, X., Xiong, Z. (2019). Mechanisms of mitigating nitrous oxide emissions from vegetable soil varied with manure, biochar and nitrification inhibitors. Agricultural and Forest Meteorology. 278, 107672. doi: 10.1016/j.agrformet.2019.107672

Duan, Y., Kong, X., Schramm, A., Labouriau, R., Eriksen, J., Petersen, S.O. (2016). Microbial N transformations and N_2_O emission after simulated grassland cultivation: Effects of the nitrification inhibitor 3,4-dimethylpyrazole phosphate (DMPP). Applied and Environmental Microbiology. 02019-16. doi:10.1128/AEM.02019-16

Di, H., Cameron, K.C., Podolyan, A., Robinson, A. (2014). Effect of soil moisture status and a nitrification inhibitor, dicyandiamide, on ammonia oxidizer and denitrifier growth and nitrous oxide emissions in a grassland soil. Soil Biology and Biochemistry. 73, 59-68. doi: 10.1016/j.soilbio.2014.02.011

Dai, Y., Di, H., Cameron, K.C., He, J. (2013). Effects of nitrogen application rate and a nitrification inhibitor dicyandiamide on ammonia oxidizers and N_2_O emissions in a grazed pasture soil. Science of the Total Environment. 465, 125-135. doi: 10.1016/j.scitotenv.2012.08.091

Elrys, A.S., Raza, S., Elnahal, A.S.M., Na, M., Ahmed, M., Zhou, J., et al. (2020). Do soil property variations affect dicyandiamide efficiency in inhibiting nitrification and minimizing carbon dioxide emissions? Ecotoxicology and Environmental Safety. 202, 110875. doi: 10.1016/j.ecoenv.2020.110875

Fan, X., Yin, C., Chen, H., Ye, M., Zhao, Y., Li, T., et al. (2019). The efficacy of 3,4-dimethylpyrazole phosphate on N_2_O emissions is linked to niche differentiation of ammonia oxidizing archaea and bacteria across four arable soils. Soil Biology and Biochemistry. 130, 82-93. doi: 10.1016/j.soilbio.2018.11.027

Fuertes-Mendizábal, T., Huérfano, X., Vega-Mas, I., Torralbo, F., Menéndez, S., Ippolito, J.A., et al. (2019). Biochar reduces the efficiency of nitrification inhibitor 3,4-dimethylpyrazole phosphate (DMPP) mitigating N_2_O emissions. Scientific Reports. 9(1), 2346. doi: 10.1038/s41598-019-38697-2

Fu, Q., Clark, I.M., Zhu, J., Hu, H., Hirsch, P.R. (2018). The short-term effects of nitrification inhibitors on the abundance and expression of ammonia and nitrite oxidizers in a long-term field experiment comparing land management. Biology and Fertility of Soils. 54, 163-172. doi

Gao, S., Guo, Y., Zhang L., Li, B., Liu, Q., Han, J. (2019). Effects of DCD and DMPP on the nitrous oxide emissions and ammonia violation from greenhouse soil under different water contents. Journal of hebei agricultural university. 42(04), 95-101. doi: 10.13320/j.cnki.jauh.2019.0084: 10.1007/s00374-017-1249-2

Guo, Y., Di, H., Cameron, K.C., Li, B. (2014). Effect of application rate of a nitrification inhibitor, dicyandiamide (DCD), on nitrification rate, and ammonia-oxidizing bacteria and archaea growth in a grazed pasture soil: An incubation study. Journal of Soils and Sediments. 14(5), 897-903. doi: 10.1007/s11368-013-0843-7

Gong, P., Zhang, L., Wu, Z., Chen, Z., Chen, L. (2013). Responses of Ammonia-Oxidizing Bacteria and Archaea in Two Agricultural Soils to Nitrification Inhibitors DCD and DMPP: A Pot Experiment. Pedosphere. 23(6), 729-739. doi:10.1016/S1002-0160(13)60065-X

Huang, Q., Wang, Q., Wu, Q., Li, S., Huang, Y., Chen, D., et al. (2019). Effects of nitrification inhibitors on nitrification and ammonia oxidizers abundance and community structure in an acidic red soil. Sugarcane and Canesugar. 2019(2), 18-26.

Hink, L., Gubry-Rangin, C., Nicol, G., Prosser, J. (2018). The consequences of niche and physiological differentiation of archaeal and bacterial ammonia oxidisers for nitrous oxide emissions. The ISME Journal. 12, 1084-1093. doi: 10.1038/s41396-017-0025-5

Kou, Y., Wei, K., Chen, G, Wang, Z., Xu, H. (2015). Effects of 3,4-dimethylpyrazole phosphate and dicyandiamide on nitrous oxide emission in a greenhouse vegetable soil. Plant, Soil and Environment. 61, 29-35. doi: 10.17221/762/2014-PSE

Li, J., Wang, S., Luo, J., Zhang, L., Wu, Z., Lindsey, S. (2021). Effects of biochar and 3,4-dimethylpyrazole phosphate (DMPP) on soil ammonia-oxidizing bacteria and nosZ-N_2_O reducers in the mitigation of N_2_O emissions from paddy soils. Journal of Soils and Sediments. 21, 1089-1098. doi: 10.1007/s11368-020-02811-z

Liu, C., Liu, H., Liu, X., Zhang, Y., Wang, L., Guan, D., et al. (2020). Nitrification inhibitor 3,4‐dimethylpyrazole phosphate (DMPP) reduces N_2_O emissions by altering the soil microbial community in a wheat-maize rotation on the North China Plain. European Journal of Soil Science. 72, 1270-1291. doi: 10.1111/ejss.13017

Li, J., Shi, Y., Luo, J., Li, Y., Wang, L., Lindsey, S. (2019a). Effects of 3,4-dimethylpyrazole phosphate (DMPP) on the abundance of ammonia oxidizers and denitrifiers in two different intensive vegetable cultivation soils. Journal of Soils and Sediments. 19, 1250-1259. doi: 10.1007/s11368-018-2155-4

Li, J., Shi, Y., Wang, L., Sun, Y., Li, Z., Wei, Z., et al. (2019b). Comparison of nitrification inhibitors on N_2_O emission and abundances of nitrifier and denitrifier in paddy soil. Journal of Plant Nutrition and Fertilizers. 25(12), 2095-2101. doi: 10.11674/zwyf.19361

Lan, T., Suter, H., Liu, R., Yuan, S., Chen, D. (2018). Effects of nitrification inhibitors on gross N nitrification rate, ammonia oxidizers, and N_2_O production under different temperatures in two pasture soils. Environmental Science and Pollution Research. 25, 28344-28354. doi: 10.1007/s11356-018-2873-6

Lan, X. (2017). Effects of organic fertilizer and DMPP on N_2_O emission and ammonia-oxidizing microorganisms. Huazhong Agricultural University.

Liu, R., Hayden, H.L., Hu, H., He, J., Suter, H., Chen, D. (2017). Effects of the nitrification inhibitor acetylene on nitrous oxide emissions and ammonia-oxidizing microorganisms of different agricultural soils under laboratory incubation conditions. Applied Soil Ecology. 119, 80-90. doi: 10.1016/j.apsoil.2017.05.034

Mao, X., Cheng, M., Xu, Q., Chen, J., Zhao, T., Yu, X., et al. (2016). Effects of nitrification inhibitors on soil N2O emission and community structure and abundance of ammonia oxidation microorganism in soil under extensively managed phyllostachys edulis stands. Acta Pedologica Sinica. 53(6), 1528-1540. doi: 10.11766/trxb201603120052

Morales, S.E., Jha, N., Saggar, S. (2015). Impact of urine and the application of the nitrification inhibitor DCD on microbial communities in dairy-grazed pasture soils. Soil Biology and Biochemistry. 88, 344-353. doi: 10.1016/j.soilbio.2015.06.009

Nair, D., Abalos, D., Philippot, L., Bru, D., Mateo-Marin, N., Petersen, S.O. (2021) Soil and temperature effects on nitrification and denitrification modified N2O mitigation by 3,4-dimethylpyrazole phosphate. Soil Biology and Biochemistry. 157. https://doi.org/10.1016/j.soilbio.2021.108224

Robinson, A., Di, H., Cameron, K. C., Podolyan, A. (2014a). Effect of soil aggregate size and dicyandiamide on N_2_O emissions and ammonia oxidizer abundance in a grazed pasture soil. Soil Use and Management. 30, 231-240. doi:10.1111/sum.12104

Robinson, A., Di, H., Cameron, K.C., Podolyan, A., He, J. (2014b). The effect of soil pH and dicyandiamide (DCD) on N_2_O emissions and ammonia oxidiser abundance in a stimulated grazed pasture soil. Journal of Soils and Sediments. 14, 1434-1444. doi: 10.1007/s11368-014-0888-2

Shi, X., Hu, H., Zhu-Barker, X., Hayden, H., Wang, J., Suter, H., et al. (2017). Nitrifier-induced denitrification is an important source of soil nitrous oxide and can be inhibited by a nitrification inhibitor 3,4-dimethylpyrazole phosphate. Environmental Microbiology. 19(12), 4851-4865. doi: 10.1111/1462-2920.13872

Shi, X., Hu, H., Müller, C., He, J., Chen, D., Suter, H.C. (2016a). Effects of the nitrification inhibitor 3,4-dimethylpyrazole phosphate on nitrification and nitrifiers in two contrasting agricultural soils. Applied and Environmental Microbiology. 82, 5236-5248. doi: 10.1128/AEM.01031-16

Shi, X., Hu, H., He, J., Chen, D., Suter, H. (2016b). Effects of 3,4-dimethylpyrazole phosphate (DMPP) on nitrification and the abundance and community composition of soil ammonia oxidizers in three land uses. Biology and Fertility of Soils. 52, 927-939. doi: 10.1007/s00374-016-1131-7

Soares, J.R., Cassman, N.A., Kielak, A.M., Pijl, A., Carmo, J.B., Lourenço, K.S., et al. (2016). Nitrous oxide emission related to ammonia-oxidizing bacteria and mitigation options from N fertilization in a tropical soil. Scientific Reports. 6, 30349. doi: 10.1038/srep30349

Shi, M., Zhang, M., Shen, F., Liang, D., Dang, H. (2011). Effects of nitrification inhibitors on nitrification inhibition and nitrite accumulation in calcareous soil. Scientia Agricultura Sinica. 44(3), 500-506. doi: 10.3864/j.issn.0578-1752.2011.03.009

Tao, R., Li, J., Hu, B., Chu, G. (2021). Mitigating N_2_O emission by synthetic inhibitors mixed with urea and cattle manure application via inhibiting ammonia-oxidizing bacteria, but not archaea, in a calcareous soil. Environmental Pollution. 273, 116478. doi: 10.1016/j.envpol.2021.116478

Torralbo, F., Menéndez, S., Barrena, I., Estavillo, J., Marino, D., González-Murua, C. (2017). Dimethyl pyrazol-based nitrifcation inhibitors effect on nitrifying and denitrifying bacteria to mitigate N_2_O emission. Scientific Reports. 7, 13810. doi: 10.1038/s41598-017-14225-y

Wang, L., Zhang G., Liu, M., Sun, S., Liu, L., Ru, S., et al. (2021). Effect of nitrification inhibitor and fertilizer reduction on reducing nitrogen source gas emission and their interaction with microbial functional genes in greenhouse vegetable field. Acta agriculturae boreali-sinica. 36(2), 196-203. doi: 10. 7668 /hbnxb. 20191952

Wang, Q., Zhang, L., Shen, J., Du, S., Han, L., He, J. (2016). Effects of dicyandiamide and acetylene on N_2_O emissions and ammonia oxidizers in a fluvo-aquic soil applied with urea. Environmental Science and Pollution Research. 23, 23023-23033. doi: 10.1007/s11356-016-7519-y

Wakelin, S.A., Clough, T.J., Gerard, E.M., O Callaghan, M. (2013). Impact of short-interval, repeat application of dicyandiamide on soil N transformation in urine patches. Agriculture, Ecosystems & Environment. 167, 60-70. doi: 10.1016/j.agee.2013.01.007

Yang, L., Zhu, G., Ju, X., Liu, R. (2021). How nitrification-related N_2_O is associated with soil ammonia oxidizers in two contrasting soils in China? Science of the Total Environment. 770, 143212. doi: 10.1016/j.scitotenv.2020.143212

Yang, J., Li, C., Xu, L., Hu, F., Li, H., Liu, M. (2013). Influence of the nitrification inhibitor DMPP on the community composition of ammonia-oxidizing bacteria at microsites with increasing distance from the fertilizer zone. Biology and Fertility of Soils. 49(1), 23-30. doi: 10.1007/s00374-012-0692-3

Yang, Y., Meng, D., Qin, H., Wu, M., Zhu, Y., Wei, W. (2012). Mechanism of nitrification inhibitor on nitrogen-transformation bacteria in vegetable soil. Acta Ecologica Sinica. 32(21), 6803-6810. doi: 10. 5846 /stxb201109231399

Zhou, X., Wang, S., Ma, S., Zheng, X., Wang, Z., Lu, C. (2020). Effects of commonly used nitrification inhibitors-dicyandiamide (DCD), 3, 4-dimethylpyrazole phosphate (DMPP), and nitrapyrin-on soil nitrogen dynamics and nitrifiers in three typical paddy soils. Geoderma. 380, 114637. doi: 10.1016/j.geoderma.2020.114637

Zhang, W., Wang, S., Xia, W., Sun, G., Liu, Z., Li, Z., et al. (2019). Effects of urease inhibitor and nitrification inhibitor on functional nitrifier and denitrifier in paddy soil. Journal of Plant Nutrition and Fertilizers. 25(6), 897-909. doi: 10.11674/zwyf.18237

Zhou, Z., Zhang, Z., Wang, M., Liu, Y., Dai, J. (2018). Effect of the nitrification inhibitor (3, 4-dimethylpyrazole phosphate) on the activities and abundances of ammonia-oxidizers and denitrifiers in a phenanthrene polluted and waterlogged soil. Ecotoxicology and Environmental Safety. 161, 474-481. doi: 10.1016/j.ecoenv.2018.06.030.

Zhang, M., Wang, W., Tang, L., Heenan, M., Xu, Z. (2018). Effects of nitrification inhibitor and herbicides on nitrification, nitrite and nitrate consumptions and nitrous oxide emission in an Australian sugarcane soil. Biology and Fertility of Soils. 54, 697-706. doi: 10.1007/s00374-018-1293-6.
